# Faded Critical Dynamics in Adult Moyamoya Disease Revealed by EEG and fMRI

**DOI:** 10.1155/2021/6640108

**Published:** 2021-04-16

**Authors:** Yu Lei, Yuzhu Li, Lianchun Yu, Longzhou Xu, Xin Zhang, Gaoxing Zheng, Liang Chen, Wei Zhang, Xiaoying Qi, Yuxiang Gu, Yuguo Yu, Ying Mao

**Affiliations:** ^1^Department of Neurosurgery, Huashan Hospital, Fudan University, Shanghai 200040, China; ^2^State Key Laboratory of Medical Neurobiology, School of Life Science and Human Phenome Institute, Institute of Brain Science, Institute of Science and Technology for Brain-Inspired Intelligence, Fudan University, Shanghai 200040, China; ^3^Institute of Theoretical Physics, Key Laboratory for Magnetism and Magnetic Materials of the Ministry of Education, Lanzhou University, Lanzhou 730000, China; ^4^Quantitative Neuroscience with Magnetic Resonance Core Center, Yale University, New Haven, CT 06520, USA

## Abstract

Criticality is considered a dynamic signature of healthy brain activity that can be measured on the short-term timescale with neural avalanches and long-term timescale with long-range temporal correlation (LRTC). It is unclear how the brain dynamics change in adult moyamoya disease (MMD). We used BOLD-fMRI for LRTC analysis from 16 hemorrhagic (*H*_MMD_) and 34 ischemic (*I*_MMD_) patients and 25 healthy controls. Afterwards, they were examined by EEG recordings in the eyes-closed (EC), eyes-open (EO), and working memory (WM) states. The EEG data of 11 *H*_MMD_ and 13 *I*_MMD_ patients and 21 healthy controls were in good quality for analysis. Regarding the 4 metrics of neural avalanches (e.g., size (*α*), duration (*β*), *κ* value, and branching parameter (*σ*)), both MMD subtypes exhibited subcritical states in the EC state. When switching to the WM state, *H*_MMD_ remained inactive, while *I*_MMD_ surpassed controls and became supercritical (*p* < 0.05). Regarding LRTC, the amplitude envelope in the EC state was more analogous to random noise in the MMD patients than in controls. During state transitions, LRTC decreased sharply in the controls but remained chaotic in the MMD individuals (*p* < 0.05). The spatial LRTC reduction distribution based on both EEG and fMRI in the EC state implied that, compared with controls, the two MMD subtypes might exhibit mutually independent but partially overlapping patterns. The regions showing decreased LRTC in both EEG and fMRI were the left supplemental motor area of *H*_MMD_ and right pre-/postcentral gyrus and right inferior temporal gyrus of *I*_MMD_. This study not only sheds light on the decayed critical dynamics of MMD in both the resting and task states for the first time but also proposes several EEG and fMRI features to identify its two subtypes.

## 1. Introduction

Moyamoya disease (MMD) is a chronic cerebrovascular disease characterized by progressive stenosis and occlusion of the terminal portion of the bilateral internal carotid arteries and their main branches. The vascular pathology leads to widespread and continuous cerebral hypoperfusion and gradual formation of compensation from collaterals as a response [1]. The blood supply of neuron activities is often disrupted, followed by less regional neural activities and cognitive impairment [2–4]. The MMD is the only known chronic cerebrovascular disease with both hemorrhagic (*H*_MMD_) and ischemic (*I*_MMD_) clinical outcomes [5, 6]. Besides the general pathophysiology mentioned above in common, the two clinical subtypes are suspected to exhibit different but unknown processes, which are proved to be irrelevant with cerebral infarct or hematoma [3, 7–9]. However, their morphological and hemodynamic differences are difficult to be discerned and rarely reported [6, 9].

Healthy brain networks in the resting state are generally characterized by well-balanced excitatory and inhibitory synaptic activities [10]. These balanced brain states may sit in a dynamic state close to the criticality. Neural networks in a critical state have been found to be efficient for information transmission and other functions [11, 12]. Such critical dynamics are associated with a sustained neural activity that exhibits scale-free avalanche distribution [13]. Cognitive tasks may shift the brain activity from the critical point to a supercritical state by activating some brain regions and suppressing others [14]. The critical dynamics can be characterized quantitatively on two timescales: neural avalanches in the short term (milliseconds) and long-range temporal correlation (LRTC) in the long term (seconds to hours) [15]. During a neuronal avalanche, spontaneous activation of one neuronal group can trigger consecutive activations of other neuronal groups within just a few milliseconds, propagating cascading waves of activity. This phenomenon has been revealed by multiple neuroimaging modalities, such as electroencephalography (EEG) and fMRI in healthy subjects [16–18]. Additionally, brains in disease states such as unconsciousness and schizophrenia exhibit faded critical dynamics [19, 20].

LRTC is another notable measurement to evaluate the critical dynamics of the neuronal system [21, 22]. Although neural oscillations present themselves with large variability in both frequency and amplitude, their fluctuations reflect a tendency toward self-organized criticality [23]. The oscillatory activity at any time is influenced by previous activities, and the LRTC is built up though local interactions. Such oscillations, reflecting short-term and long-term interactions, are observed to exist throughout the entire system. Recent studies have shown that healthy brains in the resting state are associated with a relatively large value of LRTC, while decreased LRTC has been reported in various diseased brain states such as a major depressive disorder and Alzheimer's disease [24, 25].

Clinically, rapid and accurate differential diagnosis between acute ischemia and hemorrhage is crucial for early medical and interventional treatment but the optimal time window of treatment is often missed because of its high reliance on a CT or MR scan. The MMD is expected as the promising disease template to develop a more rapid and accurate differential diagnostic tool. According to one published fMRI study of ours, a dynamic measurement of entropy was proposed as an index of the critical dynamics to describe quantitatively the spatiotemporal changes of neural communication in adult MMD [26]. It found that critical dynamics faded not only in the diseased brain but also with disease progression. Therefore, this study was performed to examine two issues as the first of its kind. One is to explore the faded critical dynamics of MMD in both the resting and task states directly through a combination of EEG (high temporal resolution) and fMRI (high spatial resolution). The other is to investigate whether the two subtypes with a similar extent of cognitive impairment exhibit different neuronal dynamics and generate several features for future rapid and bedside identification of acute cerebral ischemia and hemorrhage.

## 2. Materials and Methods

### 2.1. Participants

This study was approved by the Institutional Review Board in our hospital and conducted in accordance with the Helsinki declaration. Informed consent was signed by all the subjects of this study. From March 2017 to August 2018, 50 adult patients with MMD (16 *H*_MMD_ and 34 *I*_MMD_) were recruited. The inclusion criteria were as follows: (1) Chinese nationality, right handed, and age of over 18 years; (2) diagnosis through digital subtraction angiography, with a Suzuki grade of III or IV [1]; (3) no evidence of infarct and hematoma larger than 8 mm in the maximum dimension on structural brain images [2, 27, 28]; (4) physical ability to undergo cognitive testing and EEG tasks; (5) no severe systemic or other cerebrovascular diseases; and (6) no medical history of neurosurgery. Twenty-five healthy subjects with no cerebrovascular or mental diseases were enrolled as controls. The Mini-Mental State Examination (MMSE) was adopted for the neuropsychological evaluation of global cognitive states.

### 2.2. Data Acquisition and Preprocessing

EEG data were acquired at a sampling rate of 1000 Hz in a sound-attenuated room by using a 64-channel actiCHamp Brain Products recording system (Brain Products GmbH Inc., Munich, Germany). The impedance of all channels was below 10 K*Ω*. The experimental paradigm was presented in E-Prime 2.0, and preprocessing was performed using the MATLAB R2017b software plug-in EEGLAB 14.0.0. Data were filtered to the frequency range of 0.5–100 Hz. Then, the interference at a frequency of 50 Hz was removed using a notch filter. Independent component analysis and the ADJUST toolbox were used to remove the components of eyeblink and cardio ballistic artifacts. EEG recording was started with a 5-minute eyes-closed (EC) resting state, a 5-minute eyes-open (EO) resting state, and then 30 trials of a delayed-response working memory (WM) task for around 20 minutes and ended with a procedure composed of a 5-minute EC and a 5-minute EO resting state to examine the consistency of the data recording quality (Supplementary Figure S2). Ultimately, the EEG data from 24 patients (11 *H*_MMD_ and 13 *I*_MMD_) and 21 healthy controls were selected for good quality.

All MRI data were collected using a 3.0 Tesla scanner (GE Healthcare, GE Asian Hub, Shanghai, China) with a 32-channel intraoperative head coil. The fMRI data were acquired using gradient echo-planar imaging with the following parameters: 3.2 mm slice thickness, 2000 ms repetition time, 30 ms echo time, 90° flip angle, 220 × 220 mm^2^ field of view, 64 × 64 matrix size, and 3.438 × 3.438 × 3.2 mm^3^ voxel size. Each scan lasted for 8 min and collected 240 volumes. Preprocessing was performed using the SPM12 (https://www.fil.ion.ucl.ac.uk/spm) and DPARSF [29]. Volumes were corrected for slice timing and head motion, and subjects were excluded if their head movement exceeded 3 mm or 3 degrees. Here, we used the Friston 24-parameter model [30] to regress out the head motion effects from the realigned data (i.e., 6 head motion parameters, 6 head motion parameters on the time point before, and the 12 corresponding squared items) that were based on recent reports that higher-order models demonstrated benefits in removing head motion effects [31]. We also obtained the mean framewise displacement (FD) derived from Jenkinson's relative root mean square algorithm [32] for each participant to assess the effects of head motion on group-level statistics. After spatial normalization (Montreal Neurological Institute space), resampling (3 mm isotropic voxels), and spatial smoothing (4 mm, full-width, half-maximum Gaussian kernel), volumes were preprocessed using the linear trend subtraction and temporal filtering (0.01–0.08 Hz). In addition, the signals from the cerebrospinal fluid and white matter were regressed out to reduce the effect of nonneuronal BOLD fluctuation [33].

### 2.3. Short-Term Timescales of Critical Dynamics: Neuronal Avalanches

#### 2.3.1. Transfer of EEG Data from Amplitude to Mean Frequency

**Figure 1:**
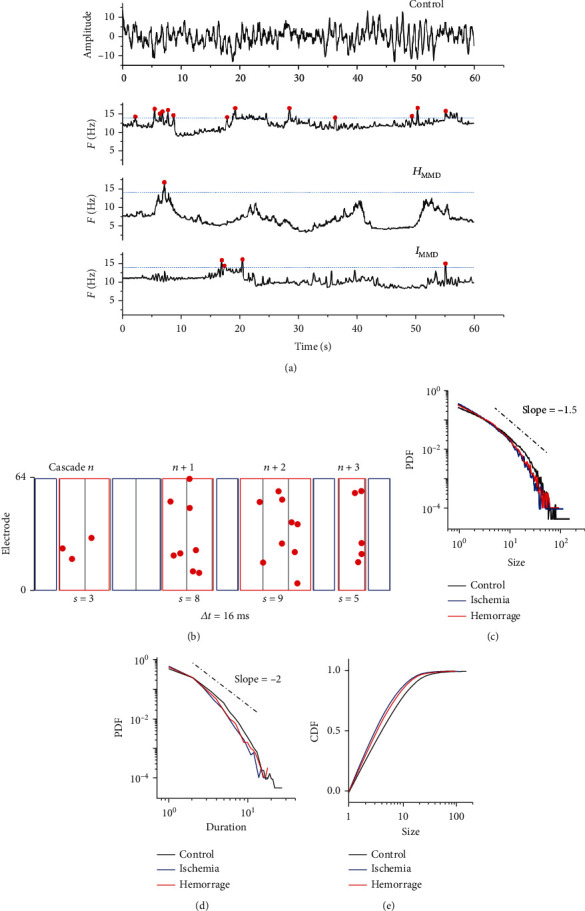
Definition and evaluation of events and avalanches in the three groups. (a) An example showing a 60-second span of EEG amplitude data from a subject and the corresponding mean frequency. (b) Segmenting data with four avalanches as examples. A cascade was defined as continuous events as time bins end with no-event time bins. The number of events in the avalanche defined the avalanche size. The number of time bins was defined as the duration. The avalanche sizes in the figure were 3, 8, 9, and 5; the durations were 2, 2, 3, and 1, respectively. (c–e) The probability density distribution (PDF) and cumulative probability density distribution (CDF) of the avalanche size (*α*) and duration (*β*) in the EC state for each of the three groups.

The time sequence of each channel was translated from amplitude to mean frequency by the short-term Fourier transform (Figures 1(a) and 1(b)). The mean frequency of each 1-sec epoch was calculated as

(1) $$\begin{array}{c} avef\left(f\right)=\frac{\sum P\left(f\right)\times f}{\sum P\left(f\right)}, \end{array}$$

with a 2 ms time shift in each channel, where *f* and *P* represent the frequency and power of the power spectrum of the signal, respectively. The average baseline of each channel was replaced with the group ensemble average of the channel to avoid noise interference caused by individual difference perturbations. Given that the EEG amplitude is mainly dominated by the low-frequency component of synchronized slow oscillations and some high-frequency activities are submerged in the slow-wave activities with high amplitudes, the EEG frequency may be a better sequence for reflecting the intensity of the neural activity rate.

#### 2.3.2. Overthreshold Event Detection and Neuronal Avalanche Extraction

For each channel, the threshold for events was defined as the mean value + 1 SD (standard deviation) of the EC state averaged overall controls. It is noted that for frequency events, 1 SD fluctuation above the mean frequency rate is strong enough to be a burst event (two and three SD were also tested, but the events were very sparse). The function “*findpeaks*” in MATLAB 2015b was used to find the peaks higher than the threshold (with a duration over 20 ms), which were defined as the events. Neuronal avalanches were defined as continuous time bins with at least one event in each channel, beginning and ending with event-free time bins. The time bin used was 16 ms (although the conclusion held true with other time bins from 4 to 20 ms). Binarized data were used for event detection.

#### 2.3.3. Calculation of the Avalanche Size (*α*) and Duration (*β*)

As shown in Figures 1(c) and 1(d), we first obtained the probability density function (PDF) of the avalanche size (number of events) and duration (number of time bins) and then fitted it to the power law distribution *P*_*α*_(*χ*) = *C*_*α*_*χ*^*α*^ using maximum likelihood estimation to obtain the slope, denoted by the size (*α*) and duration (*β*) [34, 35]. The exponent was estimated by calculating *L*(*α* | *x*) = ∏_*i*=1_^*n*^*P*_*α*_(*x*_*i*_), which means that the best-fitting exponent was calculated by maximizing the log-likelihood function. The higher absolute value of the power law exponent means the steeper fitted power law curve and indicates the higher probability of small size and duration avalanche [18]. Since the large size and duration avalanche means a rather large range and longer time of neuronal dynamics, the comparison of these parameters between the MMD and controls can be utilized to reflect the aberrant neuronal dynamics of MMD.

#### 2.3.4. Calculation of the *κ* Value

The *κ* value was used to estimate how far the system was from criticality. This nonparametric measure quantifies the difference in the subjects' cumulative density function (CDF) from the standard critical reference CDF with a power law exponent of 1.5 (Figure 1(e)) [11, 36]:

(2) $$\begin{array}{c} \kappa =1+\frac{1}{m}\overset{m}{\underset{k=1}{\sum }}\left(F^{NA}\left(\beta_{k}\right)-F\left(\beta_{k}\right)\right). \end{array}$$

The value of this parameter at approximately 1 indicates a critical state of the system, while values above or below 1 indicate the super- or subcritical states, respectively.

#### 2.3.5. Calculation of the Branching Parameter (*σ*)

The branching parameter *σ* is defined as the average number of subsequent events triggered by a single preceding event in an avalanche [34]. Thus, it was first calculated by averaging the ratio of the numbers of events in the second bin of an avalanche to the number of events in the first bin and then averaging all avalanches of a subject's time series:

(3) $$\begin{array}{c} \sigma =\frac{1}{N_{\text{av}}}\overset{N_{av}}{\underset{k=1}{\sum }}\frac{2\text{nd bin of }k^{\prime }\text{th avalanche}}{n_{\text{events}}\left(1\text{st bin of }k^{\prime }\text{th avalanche}\right)}, \end{array}$$

where *N* is the total number of cascades in the time series and *n* is the number of the events in a certain time bin. For the single-bin cascades, the branching parameter is equal to 0. Therefore, the branching parameter of each cascade can vary over a wide range and the number of the cascades must be at least 700 in this study.

### 2.4. Long-Term Timescales of Critical Dynamics: LRTC

#### 2.4.1. LRTC Based on EEG Data

Detrended fluctuation analysis was used to calculate the Hurst exponent of LRTC [37, 38]. The EEG data were bandpass filtered (finite impulse response filter) to the delta (1–4 Hz), theta (4–8 Hz), alpha (8–12 Hz), beta (12–25 Hz), and gamma (25–100 Hz) bands using a filter order of 2/minimum frequency of the band. Given a consecutive time series *y*(*t*), the first step was to subtract the mean signal and obtain the cumulative sum of the signal:

(4) $$\begin{array}{c} x\left(t\right)=\overset{t}{\underset{k=1}{\sum }}\left(y\left(k\right)-\langle y\rangle \right), \end{array}$$

where <, > denotes the time average. Then, the signal was divided into a set of time windows *W* of the same length *L* with 50% overlap. For each window in *W*, the linear trend was removed using the least-squares method. Then, we computed the average root-mean-square fluctuation, *F*(*L*), of all the time windows in *W* with an identical length *L*. The time window length was logarithmically spaced with a lower bound of 4 samples and an upper bound of 10% of the signal length [38]. According to our timescale in this work, we used 29 different window lengths *L* ranging from 100 samples to 10000 samples (0.1 ~ 10 s). The Hurst exponent *H* was defined as the coefficient of the linear regression of the sequence {*R*(*n*)/*S*(*n*)} plotted on a log-log scale. For a scale-free signal *F*(*L*) ∝ *L*^*H*^, in the case of 0 < *H* < 1, the time series was correlated. When *H* ranged from 0.5 to 1, the signal was considered to exhibit positive autocorrelation, while *H* = 0.5 indicated an uncorrelated signal.

#### 2.4.2. LRTC Based on fMRI Data

The voxel-wise Hurst exponent was adopted to evaluate the LRTC of fMRI data through the classical rescaled range (R/S) analysis [39]. The BOLD signal time series was divided into multiple shorter time series (length, *n*). The number of subtime series was *M*, and *N* = *M* × *n* was the length of the full-time series. For the subtime series of length *n*, *X*_*m*_ = {*X*_*m*1_, *X*_*m*2_, ⋯, *X*_*mn*_}, where *m* = 1,2,3, ⋯, *M*, we calculated the rescaled range *R*(*n*)/*S*(*n*):

(5) $$\begin{array}{c} \frac{R\left(n\right)}{S\left(n\right)}=\frac{1}{M}\overset{M}{\underset{m=1}{\sum }}\frac{R_{mn}}{Smn}. \end{array}$$

The *R*_*mn*_ is the difference between the extremes of subtime series *X*_*m*_. *Cd* (*Xm*) is the cumulative deviation from *i* to *k* of the subtime series:

(6) $$\begin{array}{c} Cd\left(X_{m}\right)=\overset{k}{\underset{i=1}{\sum }}\left(X_{mi}-{\bar{X}}_{m}\right), \end{array}$$

where 1 ≤ *k* ≤ *n*. *R*_*mn*_ is the difference between the maximum and minimum of *Cd* (*X*_*m*_) and minimum:

(7) $$\begin{array}{c} R_{mn}=\left[\underset{1\leq k\leq n}{\max}\overset{k}{\underset{i=1}{\sum }}\left(X_{mi}-{\bar{X}}_{m}\right)-\underset{1\leq k\leq n}{\min}\overset{k}{\underset{i=1}{\sum }}\left(X_{mi}-{\bar{X}}_{m}\right)\right]. \end{array}$$

The *S*_*mn*_ is the standard deviation of subtime series *X*_*m*_:

(8) $$\begin{array}{c} S_{mn}=\sqrt{\frac{1}{n}\overset{n}{\underset{i=1}{\sum }}{\left(X_{mi}-{\bar{X}}_{m}\right)}^{2}}. \end{array}$$

Calculating all the subtime series, we obtained the sequence {*R*(*n*)/*S*(*n*)} corresponding to the sequence {*n*}, where *n* was all the possible values of the subtime series length.

### 2.5. Statistical Analysis

For EEG data analysis, one-way ANOVA was used and all the pairwise comparisons were assessed using Tukey-Kramer's multiple comparison method. For fMRI data analysis, one-sample *t*-tests were first performed on individual Hurst exponent maps for each group in a voxel-wise manner and to explore the within-group LRTC patterns using Rest version 1.8 (*Z* > 3.48 for voxel-wise *p* < 0.001 and cluster-wise *p* < 0.05, corrected through Gaussian random field theory) [40]. Afterwards, the averaged whole-brain Hurst exponent values were generated and compared among the three groups using the one-way ANOVA. Then, two-sample *t*-tests were performed between each pair of subgroups on individual Hurst exponent maps (*Z* > 3.29 for voxel-wise *p* < 0.001 and cluster-wise *p* < 0.05).

## 3. Results

### 3.1. Clinical Information

Table 1 shows the clinical information of the involved subjects. The demographic differences among the three groups were not significant (*p* > 0.05). However, the mean MMSE score of the healthy controls was significantly higher than that of the patient groups (*p* < 0.001). Among the subjects who were selected for the EEG analysis, differences of the demographics and cognitive and task performances among the three groups were not significant (*p* > 0.05).

### 3.2. Faded Critical Dynamics of Neuronal Avalanches

#### 3.2.1. Cascade Size (*α*) and Duration (*β*)

**Figure 2:**
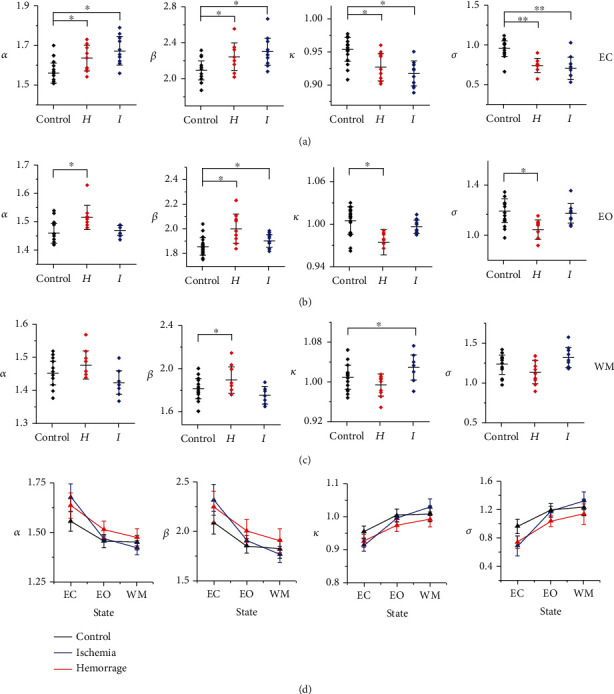
Differences in critical dynamics among the three groups in both the resting and working memory states. Rows (a–c) show the differences in neuronal avalanches among the three groups in the EC, EO, and WM states, respectively. Row (d) exhibits how critical dynamics transfer from different states in the three groups. ∗ indicates *p* < 0.05, while ∗∗ indicates *p* < 0.01.

Figure 2 indicates that the three groups exhibited significant differences in both *α* (EC, *F* = 14.11, *p* = 2.6 × 10^−5^; EO, *F* = 68.06, *p* = 2.75 × 10^−13^; and WM, *F* = 5.07, *p* = 0.0115) and *β* (EC, *F* = 9.88, *p* = 0.0004; EO, *F* = 10.37, *p* = 0.0003; and WM, *F* = 5.08, *p* = 0.0114). In the subgroup analysis, *H*_MMD_ and *I*_MMD_ exhibited significant differences in *α* in the EO (${\bar{\alpha }}_{H}=1.51\pm 0.01,$${\bar{\alpha }}_{I}=1.47\pm 0.005,$*p* = 1.03 × 10^−9^) and WM (${\bar{\alpha }}_{H}=1.48\pm 0.01,$${\bar{\alpha }}_{I}=1.42\pm 0.01$, *p* = 0.0082) states and in *β* in the WM state (${\bar{\beta }}_{H}=1.90\pm 0.04,$${\bar{\beta }}_{I}=1.77\pm 0.03$, *p* = 0.0088). The control and *I*_MMD_ groups showed significant differences in both *α* (${\bar{\alpha }}_{C}=2.08\pm 0.02,$${\bar{\alpha }}_{I}=1.68\pm 0.02$, *p* = 3.35 × 10^−5^) and *β* (${\bar{\beta }}_{C}=2.08\pm 0.02,$${\bar{\beta }}_{I}=2.31\pm 0.04$, *p* = 5.23 × 10^−4^) in the EC state. Additionally, the control and *H*_MMD_ groups exhibited significant differences in *α* in both the EC (${\bar{\alpha }}_{C}=2.08\pm 0.02$, ${\bar{\alpha }}_{H}=1.63\pm 0.02$, *p* = 0.0059) and EO (${\bar{\alpha }}_{C}=1.46\pm 0.007,{\bar{\alpha }}_{H}=1.51\pm 0.01$, *p* = 9.57 × 10^−10^) states and in *β* in the EO state (${\bar{\beta }}_{C}=1.85\pm 0.01,$${\bar{\beta }}_{H}=2.00\pm 0.04$, *p* = 1.53 × 10^−4^).

#### 3.2.2. Deviations from Criticality with *κ* and the Branching Parameter of *σ*

The three groups exhibited significant differences in both *κ* (EC, *F* = 15.51, *p* = 1.18 × 10^−5^; EO, *F* = 66.34, *p* = 4.02 × 10^−13^; and WM, *F* = 5.85, *p* = 0.0063) and *σ* (EC, *F* = 23.01, *p* = 2.84 × 10^−7^; EO, *F* = 11.35, *p* = 0.0001; and WM, *F* = 5.33, *p* = 0.0094) in all three states. *H*_MMD_ and *I*_MMD_ showed significant differences in *κ* in the EO (${\bar{\kappa }}_{H}=0.97\pm 0.006,$${\bar{\kappa }}_{I}=0.99\pm 0.003,$*p* = 1.26 × 10^−9^) and WM (${\bar{\kappa }}_{H}=0.99\pm 0.007,$${\bar{\kappa }}_{I}=1.03\pm 0.008$, *p* = 0.0044) states and in *σ* in the WM state (${\bar{\sigma }}_{H}=1.14\pm 0.045,$${\bar{\sigma }}_{I}=1.32\pm 0.04$, *p* = 0.0027). The control and *I*_MMD_ groups showed significant differences in both *κ* (${\bar{\kappa }}_{C}=0.95\pm 0.004,$${\bar{\kappa }}_{I}=0.91\pm 0.005$, *p* = 2.03 × 10^−5^) and *σ* (${\bar{\sigma }}_{C}=0.96\pm 0.21,$${\bar{\sigma }}_{I}=0.70\pm 0.005$, *p* = 1.43 × 10^−6^) in the EC state. Additionally, the control and *H*_MMD_ showed significant differences in *κ* in both the EC (${\bar{\kappa }}_{C}=0.95\pm 0.004,$${\bar{\kappa }}_{H}=0.93\pm 0.006$, *p* = 0.0022) and EO (${\bar{\kappa }}_{C}=1.01\pm 0.004,$${\bar{\kappa }}_{H}=0.97\pm 0.006,$*p* = 9.57 × 10^−10^) states and in *σ* in both the EC (${\bar{\sigma }}_{C}=0.96\pm 0.21,$${\bar{\sigma }}_{H}=0.74\pm 0.03$, *p* = 4.42 × 10^−5^) and EO (${\bar{\sigma }}_{C}=1.19\pm 0.02,$${\bar{\sigma }}_{H}=1.04\pm 0.02$, *p* = 1.13 × 10^−4^) states. The results kept the same tendency when utilizing a different parameter to calculate.

#### 3.2.3. State Transition

The three groups exhibited a similar transition tendency between different states in all parameters (Figure 2(d)). For example, considering *α*, both subtypes of MMD presented with larger values than controls in the EC state, implying a subcritical state. Similarly, MMD exhibited less activity than controls when switching to the EO state. Interestingly, when changing into the WM state, the *H*_MMD_ group remained inactive but the *I*_MMD_ group exhibited supercritical dynamics that were even more active than those of the controls.

### 3.3. Faded Critical Dynamics of LRTC

#### 3.3.1. LRTC Based on EEG Data

**Figure 3:**
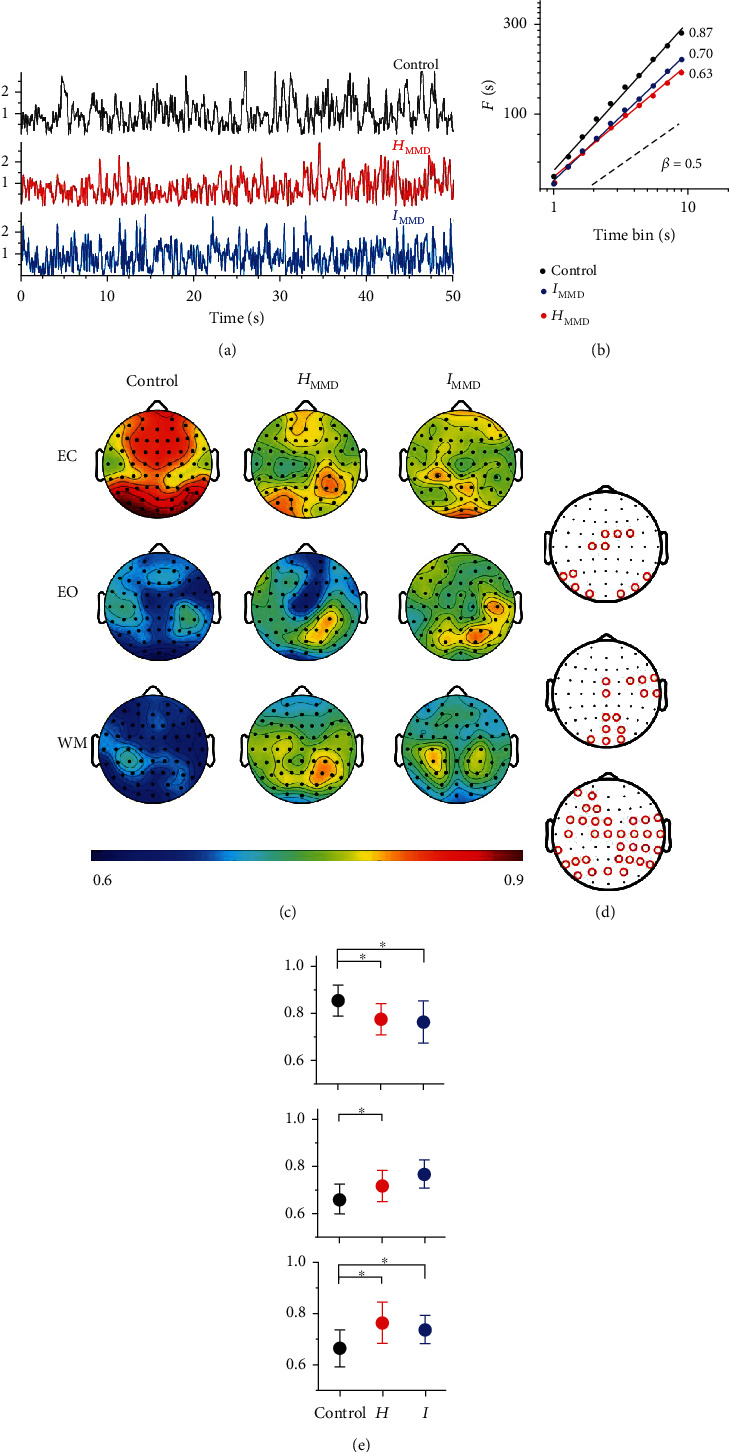
Hurst exponent mapping of the three groups with reference to the alpha band signal. In order to characterize the EEG amplitude dynamics of alpha oscillation, the data were bandpass filtered from 8 to 13 Hz and the amplitude envelope of the oscillations was extracted using the Hilbert transform. (a) Shows the amplitude envelope of the three groups in the EC state. The controls exhibit a regular oscillation, while the patients tend to present with a more random and rapidly changing amplitude. (b) Shows the fluctuation function of different time bin lengths from 1 to 10 seconds; the slope is defined as the Hurst exponent and represents the long-range temporal correlation. Regarding the slope, the white noise is equal to 0.5, while the controls reached 0.87. (c) The Hurst exponents of the three groups were mapped in the resting and WM states and are presented in rows 1 to 3. (d) The channels marked with red circles are those that show significant differences among the three groups in the EC, EO, and WM states, respectively. (e) The mean Hurst exponent value of the generated channels in Figure 3(d) was calculated and compared among the three groups. ∗ indicates *p* < 0.05.

The alpha band signal was taken for Hurst exponent analysis.  Figure 3(a) indicates that the amplitude envelopes of the MMD groups were more analogous to the random noise than the control amplitude envelope was (the EC state, with a central-parietal channel as an example). The averaged Hurst exponents of the *H*_MMD_ and *I*_MMD_ groups were approximately 0.63 and 0.70, respectively, while that of the controls was 0.87 (Figure 3(b)).

The Hurst exponents of all the recording channels were calculated and mapped in Figure 3(c). Then, channels with significant differences in Hurst exponents among the three groups were mapped (Figure 3(d), Supplementary Table S1, ANOVA, *p* < 0.05). Afterwards, the mean Hurst exponent value of the generated channels was calculated and compared among the three groups (Figure 3(e)). In the EC state, the control group exhibited significantly higher values $\left({\bar{H}}_{C}=0.85\pm 0.02\right)$ in these channels than either *H*_MMD_ (${\bar{H}}_{H}=0.78\pm 0.02$, *p* = 0.0089) or *I*_MMD_ (${\bar{H}}_{I}=0.76\pm 0.02$, *p* = 0.005). In the EO state, more channels presented a significant group difference and the *I*_MMD_ showed a significantly higher value $\left({\bar{H}}_{I}=0.76\pm 0.01\right)$ than the controls $\left({\bar{H}}_{C}=0.66\pm 0.02\right)$ in these channels (*p* = 0.0062). Furthermore, even more channels showed significant group differences in Hurst exponents in the WM state and the controls showed significantly lower values $\left({\bar{H}}_{C}=0.66\pm 0.02\right)$ than the *H*_MMD_ group (${\bar{H}}_{H}=0.76\pm 0.02$, *p* = 0.0019) or the *I*_MMD_ group (${\bar{H}}_{I}=0.74\pm 0.02$, *p* = 0.0080).

**Figure 4:**
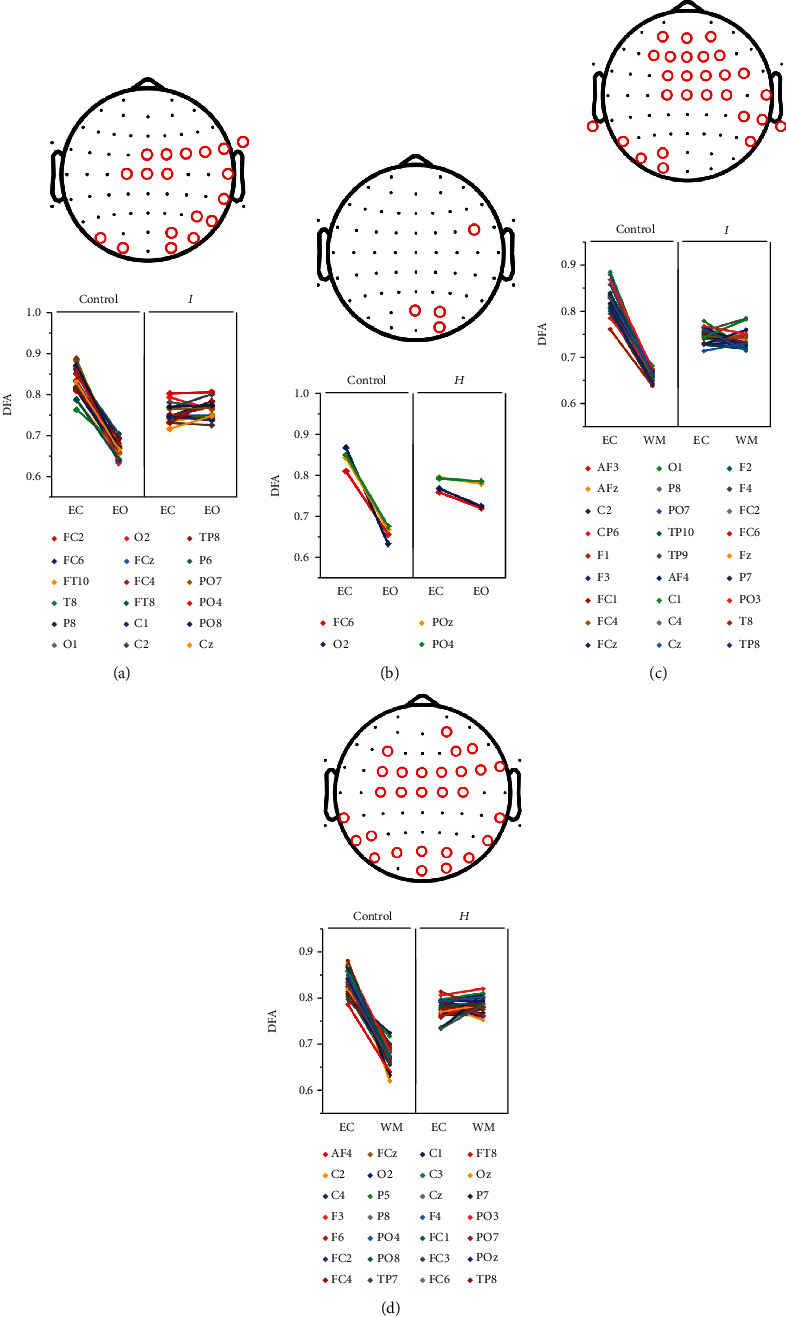
Comparison of long-range temporal correlation between controls and subtypes of MMD separately under state transition. Channels showing significant differences between controls and moyamoya subtypes in Hurst exponent changes during state transition are marked in red in the upper panel and listed in the lower panel. *I*: *I*_MMD_; *H*: *H*_MMD_; DFA: detrended fluctuation analysis.

When switching from the EC to EO state, 18 channels had significant Hurst exponent changes in the *I*_MMD_ group compared to the control group (Figures 4(a), *p* < 0.05). Interestingly, the Hurst exponents of these 18 channels all decreased in the controls, while the *I*_MMD_ group exhibited a chaotic pattern of change in these channels. In comparison, only 4 channels showed significant changes in the *H*_MMD_ group compared to controls (Figure 4(b), *p* < 0.05). Similarly, the Hurst exponents of these 4 channels all decreased in the controls, while the *H*_MMD_ also showed a chaotic changing pattern in these channels. When switching from the EC to WM state, 27 channels in the *I*_MMD_ and 28 channels in the *H*_MMD_ showed significant changes in their Hurst exponents compared to those in the controls (Figures 4(c) and 4(d), *p* < 0.05). Similarly, all these channels exhibited a large decline in Hurst exponents in the controls, while presenting with no regular pattern of change in either *I*_MMD_ or *H*_MMD_.

#### 3.3.2. LRTC Based on fMRI Data

**Figure 5:**
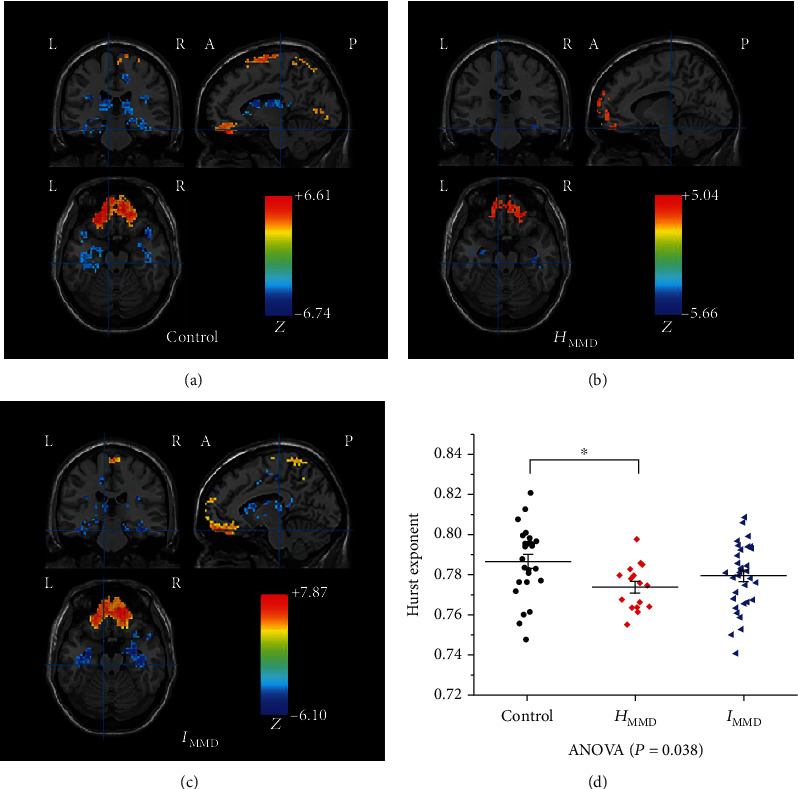
Long-range temporal correlation patterns of the three groups. The Hurst exponent patterns of the controls (a), *H*_MMD_ (b), and *I*_MMD_ (c) are presented. The statistical threshold was set at *Z* > 3.48 for voxel-wise *p* < 0.0005. ANOVA was used to detect the difference in the mean Hurst exponent value among the three groups (d). R: right; L: left; P: posterior; A: anterior. ∗ indicates a significant difference in subgroup analysis.

The Hurst exponent patterns of the three groups are presented in Figures 5(a)–5(c). Visual inspection indicated that in all three groups, the bilateral orbital frontal gyrus (OFG) and left precuneus (PCu) exhibited high values, while the bilateral fusiform gyrus (FFG), left inferior temporal gyrus(ITG), and left insular gyri (IG) showed low values. In addition, the bilateral supplemental motor area (SMA), precentral gyrus (PreCG), and postcentral (PoCG) gyrus of the controls; the left medial superior frontal gyrus (SFGmed) and right PCu of *H*_MMD_ patients; and the bilateral SFGmed, dorsolateral prefrontal gyrus (DLPFC), SMA, and right PCu of *I*_MMD_ patients all exhibited high values. In addition, the bilateral caudate nucleus (CN), hippocampus (HIP), parahippocampal gyrus (PHG), and thalamus (THA) of both controls and *I*_MMD_ showed low values. Similar to the EEG results in the EC state (Figure 3(e)), the controls exhibited the highest mean Hurst exponent value among the three groups (Figure 5(d), ANOVA, *F* = 3.397, *p* = 0.038), showing as controls (0.786 ± 0.018) > *I*_MMD_ (0.779 ± 0.016) > *H*_MMD_ (0.774 ± 0.011).

**Figure 6:**
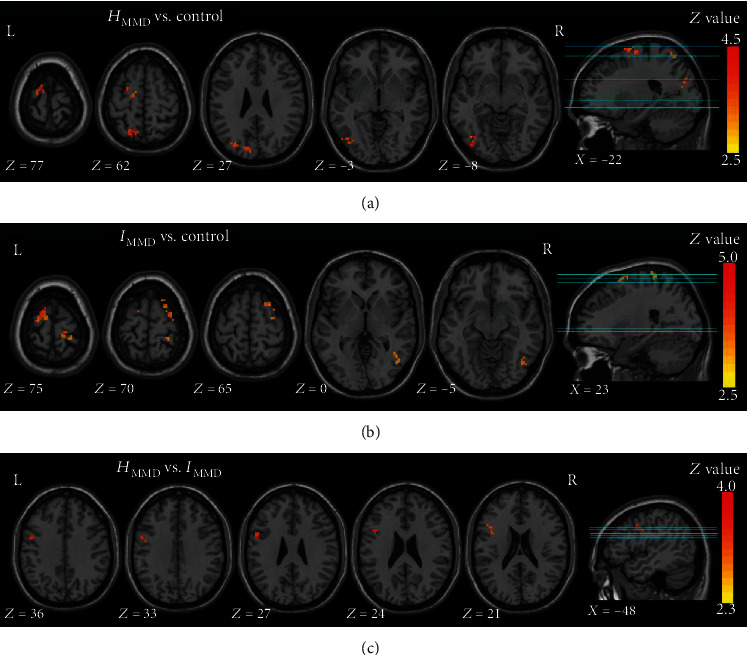
Regional LRTC differences among the three groups. The control group exhibited significantly higher Hurst exponent values than the *H*_MMD_ (a) and *I*_MMD_ groups (b) in regions with light-colored markers. The *I*_MMD_ showed significantly higher values than the *I*_MMD_ (c) in regions with light-colored markers. The statistical threshold was set at *z* > 3.29 for a voxel-wise *p* threshold of 0.001.

Compared with controls, significant decreases in the Hurst exponent in the *H*_MMD_ group were found in the left SMA, left DLPFC, left PCu, left superior parietal gyrus (SPG), and left middle occipital gyrus (MOG). Afterwards, significant decreases in the *I*_MMD_ group compared to controls were found in the bilateral DLPFC, left SMA, right PreCG, right PoCG, and right ITG. No regions in MMD exhibited significantly higher Hurst exponent values than the controls. In addition, the left PreCG exhibited a significantly lower value in *H*_MMD_ than in *I*_MMD_ but no region showed a significant difference in the opposite direction (Figure 6, Table 2).

Since head micromovements could introduce artefactual interindividual differences in resting-state fMRI metrics [41, 42], we also measured the difference of head motion among the three groups. Although the *I*_MMD_ exhibited the highest FD (Welch's ANOVA, *p* = 0.045, Supplementary Figure S5), the mean Hurst exponent was not correlated significantly with mean FD across participants (*p* > 0.05, Supplementary Figure S6). The impact of head motion on the group-level differences was then tested by adding mean FD as a covariate, and results confirmed that head motion was not responsible for the differences among the three groups (Supplementary Figure S7 and Table S2).

#### 3.3.3. LRTC Colocalization Patterns Based on EEG and fMRI Data in the EC State

**Figure 7:**
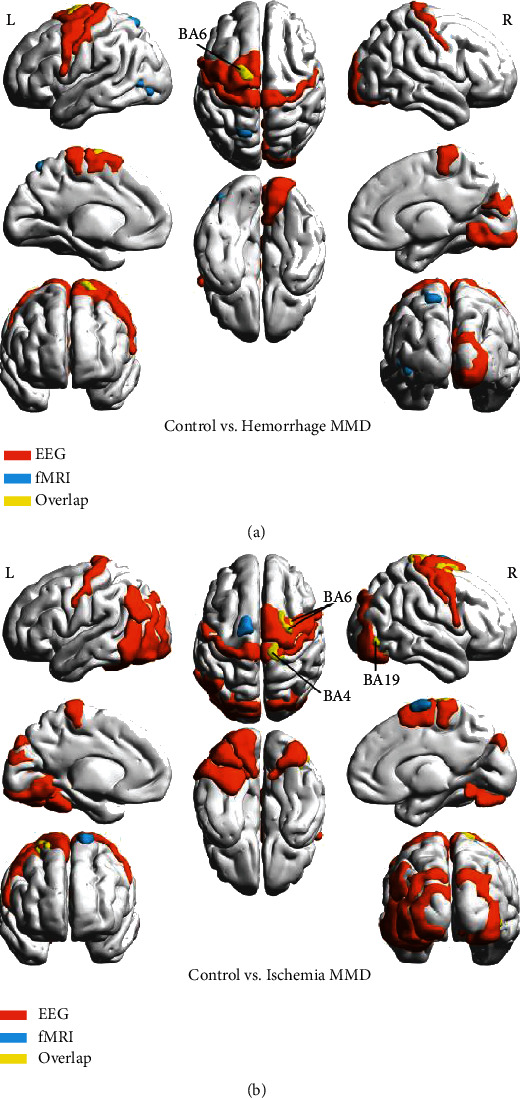
Neuroanatomical visualization showing the LRTC pattern colocalization on EEG and fMRI in MMD as compared to controls. The EEG channel positions with significantly different Hurst exponents between MMD subtypes and controls are projected onto a Brodmann's area template based on a published atlas. For *H*_MMD_ (a), only the left BA6 overlapped with the voxel-wise fMRI pattern. For *I*_MMD_ (b), only the right BA4, right BA6, and right BA19 overlapped with the fMRI pattern. The EEG channel positions are marked in orange, the fMRI positions are marked in blue, and the overlapping areas are marked in yellow. BA: Brodmann's area; R: right; L: left.

The EEG channel placement was projected onto the cortical surface and converted to the Talairach Stereotactic System based on a published Brodmann's area (BA) atlas [43]. Afterwards, we assessed the colocalization of regions with LRTC abnormities based on EEG data and fMRI data in the EC state. Compared to controls, regions with a significant Hurst exponent decrease in the *H*_MMD_ group based on overlapping data were found in the left SMA (BA6, Figure 7(a)). In addition, regions with a significant decrease in the *I*_MMD_ group based on overlapping data compared to controls were found in the right PreCG and PoCG (BA4 and BA6) and right ITG (BA19, Figure 7(b)).

## 4. Discussion

The criticality theory provides a novel insight into the neuronal dynamics underlying brain disorders. This study was the first to apply multiscale critical dynamics analysis to examine multimodal dynamical features in two moyamoya subtypes as compared to healthy controls. The neuronal avalanches on both fast and slow timescales were analyzed during rest and task performance, and several critical EEG features were derived. Both hemorrhagic and ischemic MMD exhibited particularly low EEG frequency activity and distinct subcritical dynamics, which could be distinguished easily from those of healthy controls. In addition, the decreased long-term correlations revealed in both high temporal (EEG) and spatial (fMRI) resolution were observed to reflect distinct neurophysiological processes associated with abnormal vascular network patterns in hemorrhagic and ischemic brains. Besides, this study provided clues for further rapid differential diagnosis between acute stroke and hemorrhage at the very early phase by use of EEG instead of CT and MR, which had greater advantages of rapidness, convenience, low cost, and radiation safe. Undoubtedly, time is the brain in treatment of acute ischemic stroke [44].

Previous investigations have suggested that the healthy brain in the resting state is usually characterized by well-balanced excitatory and inhibitory synaptic activities. These balanced levels of excitation and inhibition drive irregular spontaneous firing activities that exhibit scale-free avalanche distributions in the brain. Such a scale-free state can be effectively described by criticality [13]. Any input stimulus could effectively drive the brain into a supercritical state with additional excitatory activity, while relaxed low-signal states such as sleep can slow down the activity of the brain and shift it into a subcritical state. We noted that both subtypes of MMD exhibited subcritical states in the EC state and these suppressed dynamics prevented adaptive switching of brain function from introspective to extrospective states. This phenomenon might result from serious metabolic decrease and low neural activity rates caused by the chronic steno-occlusive angiopathy of MMD.

When switched to the EO state, both healthy controls and MMD presented with more neural activity and the MMD group remained less active than the controls. However, the ischemic moyamoya brains demonstrate stronger neural activities than hemorrhagic ones in the EO state. When switched to the WM state, all three groups exhibited more neural activities than that in the EO state, as was expected. Interestingly, the ischemic subtype surpassed the controls, while the hemorrhagic subtype still remained the least active. All parameters of neuronal avalanches exhibited similar results and were mutually verified. For healthy subjects, this phenomenon is reasonable because working memory is a behavioral state and requires effective neuronal activity to accomplish tasks [45]. However, we note that patients with ischemic MMD need to maintain a supercritical status to achieve similar scores to patients with hemorrhagic MMD and healthy subjects in certain WM tasks. In addition, these EEG features of neuronal avalanches during tasks may provide valuable clues for understanding the different neurophysiological processes of *I*_MMD_ and *H*_MMD_.

For healthy brains in the EC state, spatially distributed neuronal activity may oscillate in phase with each other and result in high LRTC values [46–48]. Compared with controls, the MMD group exhibited a reduced LRTC value close to 0.5 in the EC state, implying less correlated and more random brain activity. When subjects switched to EO and WM states, however, the LRTC value decreased sharply in the controls but both the *I*_MMD_ and *H*_MMD_ groups remained chaotic. The controls exhibited the lowest LRTC value in both the EO and WM states. In addition, spatial patterns of LRTC differences among the three groups were mapped based on EEG channels in both resting and WM states and under state transition. The healthy brain breaks the overall non-task-related dynamic balance into a set of subnetworks, such as the default-mode network, to deal with input signals effectively during state transition [49, 50]. However, the abnormal LRTC values and distribution of MMD in this study imply a completely different breakdown process for long-range neuronal fluctuations.

To further locate the regions with significant LRTC abnormities in MMD, we also examined BOLD fluctuations on fMRI due to the high spatial resolution of this modality. The results indicate that in the EC state, the patterns of the LRTC decreases in the two moyamoya subtypes are mutually independent but overlap in the left DLPFC of the executive control network and the left SMA of the salience network. Nevertheless, all regions of these patterns are key nodes involved in planning or direct control of movement, language, and visual information [51]. Referring to the pathophysiological nature of MMD, chronic stenosis/occlusion of the anterior circulation (bilateral internal carotid arteries and their main branches) is often followed by the collaterals from bilateral external carotid arteries and posterior circulation (bilateral vertebrobasilar arteries). Thus, the mismatch of the anterior circulation degradation and collateral development often results in a seemingly random and individualized cerebral hypoperfusion [7, 27, 52]. However, previous fMRI studies of MMD all revealed that patterns of functional deterioration are not random and key nodes of brain network such as the DLPFC, left SMA, are always involved [3, 4, 26, 28, 53]. Thus, this paper provides a crucial evidence that to output a similar extent of cognitive impairment, the neurophysiological processes of the two moyamoya subtypes may be mutually independent but overlap in some key nodes of the brain networks. Furthermore, we wondered whether there were potential links between spatial delay and temporal decay of neuronal oscillations in MMD and we attempted to trace their anatomical basis through both EEG and fMRI in the EC state. The identified regions are believed to play key roles in the neurophysiological processes of cognitive impairment in both ischemic and hemorrhagic MMD.

This study has several limitations that must be addressed. First, the EEG and fMRI data were not acquired at the same time. In order to generate a more stable and reliable result, the simultaneous EEG-fMRI technology should be used in future studies. Second, the study is based on a small sample size because completing EEG tasks is difficult for moyamoya patients with executive dysfunction. More patients are in need not only to increase the statistical power but to involve patients with Suzuki grading I–II and V–VI so as to obtain more knowledge of disease progression. Nevertheless, this study is the first of its kind to characterize the variability of brain dynamics in MMD on both short-term and long-term timescales and to show different neurophysiological features of its hemorrhagic and ischemic subtypes.

## Figures and Tables

**Table 1:** Demographic features of the 3 groups.

Index	*H*_MMD_	*I*_MMD_	Controls	*F*/*χ*^2^ value (*p* value)
fMRI cohort				
Subjects, *n*	16	34	25	/
Age (years)	42.31 ± 7.87	40.41 ± 9.71	41.32 ± 11.38	0.815 (0.309)
Male (%)	6 (37.5)	16 (47.1)	12 (48.0)	0.509 (0.775)
Education (years)	9.06 ± 5.13	9.38 ± 3.99	8.96 ± 4.37	0.729 (0.694)
MMSE	22.75 ± 4.86	25.44 ± 3.82	28.2 ± 1.71	22.076 (<0.001)
EEG cohort				
Subjects, *n*	11	13	21	/
Age (years)	40.91 ± 9.58	41.23 ± 14.60	43.38 ± 9.06	0.326 (0.858)
Male (%)	4 (36.4)	5 (38.5)	12 (57.1)	2.057 (0.357)
Education (years)	8.84 ± 2.60	8.72 ± 2.45	8.33 ± 2.39	0.408 (0.815)
MMSE	24.92 ± 1.44	24.82 ± 1.17	25.16 ± 2.39	2.305 (0.316)
WM accuracy	0.773 ± 0.217	0.759 ± 0.150	0.827 ± 0.146	0.670 (0.7165)

*H*_MMD_: hemorrhagic moyamoya disease; *I*_MMD_: ischemic moyamoya disease; MMSE: Mini-Mental State Examination.

**Table 2:** Regional LRTC differences between each pair of the three groups.

Brain regions			MNI coordinates (mm)			
	BA	Vol (mm^3^)	*x*	*y*	*z*	Maximum *Z*
*H*_MMD_ vs controls						
Left MOG	19/39/37	783	−30	−75	24	4.627
Left SMA	6	567	−12	−6	78	4.533
Left PCu	7/5	324	−15	−63	63	4.389
Left SPG	5/7	405	−18	−63	63	4.402
Left DLPFC	6	918	−15	−3	78	4.311
*I*_MMD_ vs controls						
Left DLPFC	6	675	−18	−6	78	5.058
Left SMA	6	459	−12	0	78	4.808
Right PoCG	4/3	540	15	−30	75	4.606
Right DLPFC	6/8	972	33	0	63	4.458
Right ITG	19/37	702	48	−69	−6	4.232
Right PreCG	4/6	405	15	−27	75	4.137
*H*_MMD_ vs *I*_MMD_						
Left PreCG	6	540	−51	3	27	4.156

The *x*, *y*, and *z* coordinates represent the primary peak location in the MNI space. BA: Brodmann's area; SMA: supplementary motor area; DLPFC: dorsolateral prefrontal gyrus; MNI: Montreal Neurological Institute; MOG: middle occipital gyrus; PCu: precuneus; SPG: superior parietal gyrus; PoCG: postcentral gyrus; ITG: inferior temporal gyrus; PreCG: precentral gyrus.

## Data Availability

The data used to support the findings of this study are available from the corresponding author upon request.

## Supplementary Materials

Supplementary Materials Figure S1: the schematic instruction of the process of EEG data. EEG data was preprocessed and then transferred to mean frequency data to extract the cascade through event detection. The cascade size and duration were qualified to provide critical information used for clinical diagnosis. The artifact-free data was also bandpass filtered to measure the long-range temporal correlation altered in the patients. Figure S2: the EEG recording procedure. The EEG recording was started with a 5-minute eyes-closed (EC), a 5-minute eyes-open (EO) resting state, and then 30 trials of a delayed-response working memory (WM) task for around 20 minutes and ended with a procedure composed of a 5-minute EC and a 5-minute EO resting state to examine the consistency of the data recording quality. Figure S3: abnormal functional network connectivity mode of the three groups (alpha band). WM: working memory. Figure S4: Difference of dynamic (left) and static (right) mean frequencies with nonoverlapping 10 seconds' epoch of EEG data among the three groups of hemorrhagic MMD, ischemic MMD, and healthy control in the resting and working memory states. Figure S5: differences of head motion among the three groups. The mean framewise displacement (FD) derived from Jenkinson's relative root mean square (RMS) algorithm [32] was used to measure the extent of head motion. The *I*_MMD_ groups exhibited the highest FD (Welch's ANOVA, *p* = 0.045): (*I*_MMD_(0.064 ± 0.035) > control(0.057 ± 0.017) > *H*_MMD_(0.0478 ± 0.014)). Figure S6: the correlation between head motion and Hurst exponent. Red lines indicate the linear fit. Figure S7: between-group difference of LRTC maps with the consideration of head motion effects. A, the *z*-statistical difference maps of LRTC between the healthy and hemorrhagic MMD groups; the positive value is termed as the stronger effect in controls. The statistical threshold is set as *z* > 3.29 for a voxel-wise *p* threshold of 0.001; the minimum cluster size for a voxel *p* threshold of 0.001 and cluster *p* threshold of 0.05 is 24 voxels. B, the *z*-statistical difference maps of LRTC between the healthy and ischemic MMD groups; the positive value means a stronger effect in controls. The statistical threshold is set at *z* > 3.29 for a voxel-wise *p* threshold of 0.001; the minimum cluster size for a voxel *p* threshold of 0.001 and cluster *p* threshold of 0.05 is 21 voxels. C, the *z*-statistical difference maps of LRTC between the ischemic and hemorrhagic MMD groups; the positive value indicates a stronger effect in ischemic MMD. The statistical threshold is set at *z* > 2.97 for a voxel-wise *p* threshold of 0.003; the minimum cluster size for a voxel *p* threshold of 0.001 and a cluster *p* threshold of 0.05 is 29 voxels. R: right; L: left. Table S1: channels with significant difference of Hurst exponent among the three groups in all three states. Table S2: comparison of brain regions with significant difference in LRTC among groups with and without framewise displacement covariate.

## References

[1] Suzuki J., Takaku A. (1969). Cerebrovascular “moyamoya” disease. Disease showing abnormal net-like vessels in base of brain. *Archives of Neurology*.

[2] Su S. H., Hai J., Zhang L., Yu F., Wu Y. F. (2013). Assessment of cognitive function in adult patients with hemorrhagic moyamoya disease who received no surgical revascularization. *European Journal of Neurology*.

[3] Lei Y., Li Y., Ni W. (2014). Spontaneous brain activity in adult patients with moyamoya disease: a resting-state fMRI study. *Brain Research*.

[4] Kazumata K., Tha K. K., Narita H. (2015). Chronic ischemia alters brain microstructural integrity and cognitive performance in adult moyamoya disease. *Stroke*.

[5] Burke G. M., Burke A. M., Sherma A. K., Hurley M. C., Batjer H. H., Bendok B. R. (2009). Moyamoya disease: a summary. *Neurosurgical Focus*.

[6] Scott R. M., Smith E. R. (2009). Moyamoya disease and moyamoya syndrome. *The New England Journal of Medicine*.

[7] Lei Y., Li Y. J., Guo Q. H. (2017). Postoperative executive function in adult moyamoya disease: a preliminary study of its functional anatomy and behavioral correlates. *Journal of Neurosurgery*.

[8] Kang S., Liu X., Zhang D. (2019). Natural course of moyamoya disease in patients with prior hemorrhagic stroke. *Stroke*.

[9] Zhang M., Tang J., Liu N., Xue Y., Ren X., Fu J. (2020). Postoperative functional outcomes and prognostic factors in two types of adult moyamoya diseases. *Journal of Stroke and Cerebrovascular Diseases*.

[10] Poil S. S., Hardstone R., Mansvelder H. D., Linkenkaer-Hansen K. (2012). Critical-state dynamics of avalanches and oscillations jointly emerge from balanced excitation/inhibition in neuronal networks. *The Journal of Neuroscience*.

[11] Shew W. L., Yang H., Petermann T., Roy R., Plenz D. (2009). Neuronal avalanches imply maximum dynamic range in cortical networks at criticality. *The Journal of Neuroscience*.

[12] Shew W. L., Yang H., Yu S., Roy R., Plenz D. (2011). Information capacity and transmission are maximized in balanced cortical networks with neuronal avalanches. *The Journal of Neuroscience*.

[13] Shriki O., Alstott J., Carver F. (2013). Neuronal avalanches in the resting MEG of the human brain. *The Journal of Neuroscience*.

[14] Fagerholm E. D., Lorenz R., Scott G. (2015). Cascades and cognitive state: focused attention incurs subcritical dynamics. *The Journal of Neuroscience*.

[15] Zhigalov A., Arnulfo G., Nobili L., Palva S., Palva J. M. (2015). Relationship of fast- and slow-timescale neuronal dynamics in human MEG and SEEG. *The Journal of Neuroscience*.

[16] Solovey G., Miller K. J., Ojemann J. G., Magnasco M. O., Cecchi G. A. (2012). Self-regulated dynamical criticality in human ECoG. *Frontiers in Integrative Neuroscience*.

[17] Tagliazucchi E., Balenzuela P., Fraiman D., Chialvo D. R. (2012). Criticality in large-scale brain FMRI dynamics unveiled by a novel point process analysis. *Frontiers in Physiology*.

[18] Cocchi L., Gollo L. L., Zalesky A., Breakspear M. (2017). Criticality in the brain: a synthesis of neurobiology, models and cognition. *Progress in Neurobiology*.

[19] Meisel C., Olbrich E., Shriki O., Achermann P. (2013). Fading signatures of critical brain dynamics during sustained wakefulness in humans. *The Journal of Neuroscience*.

[20] Yang G. J., Murray J. D., Repovs G. (2014). Altered global brain signal in schizophrenia. *Proceedings of the National Academy of Sciences of the United States of America*.

[21] Linkenkaer-Hansen K., Nikouline V. V., Palva J. M., IImoniemi R. J. (2001). Long-range temporal correlations and scaling behavior in human brain oscillations. *The Journal of Neuroscience*.

[22] Linkenkaer-Hansen K., Smit D. J., Barkil A. (2007). Genetic contributions to long-range temporal correlations in ongoing oscillations. *The Journal of Neuroscience*.

[23] Nikulin V. V., Brismar T. (2005). Long-range temporal correlations in electroencephalographic oscillations: relation to topography, frequency band, age and gender. *Neuroscience*.

[24] Linkenkaer-Hansen K., Monto S., Rytsälä H., Suominen K., Isometsä E., Kähkönen S. (2005). Breakdown of long-range temporal correlations in theta oscillations in patients with major depressive disorder. *The Journal of Neuroscience*.

[25] Montez T., Poil S.-S., Jones B. F. (2009). Altered temporal correlations in parietal alpha and prefrontal theta oscillations in early-stage Alzheimer disease. *Proceedings of the National Academy of Sciences of the United States of America*.

[26] Lei Y., Song B., Chen L. (2020). Reconfigured functional network dynamics in adult moyamoya disease: a resting-state fMRI study. *Brain Imaging and Behavior*.

[27] Karzmark P., Zeifert P. D., Bell-Stephens T. E., Steinberg G. K., Dorfman L. J. (2012). Neurocognitive impairment in adults with moyamoya disease without stroke. *Neurosurgery*.

[28] Lei Y., Su J., Jiang H. (2017). Aberrant regional homogeneity of resting-state executive control, default mode, and salience networks in adult patients with moyamoya disease. *Brain Imaging and Behavior*.

[29] Yan C. G., Wang X. D., Zuo X. N., Zang Y. F. (2016). DPABI: data processing & analysis for (resting-state) brain imaging. *Neuroinformatics*.

[30] Friston K. J., Williams S., Howard R., Frackowiak R. S., Turner R. (1996). Movement-related effects in fMRI time-series. *Magnetic Resonance in Medicine*.

[31] Yan C. G., Cheung B., Kelly C. (2013). A comprehensive assessment of regional variation in the impact of head micromovements on functional connectomics. *NeuroImage*.

[32] Jenkinson M., Bannister P., Brady M., Smith S. (2002). Improved optimization for the robust and accurate linear registration and motion correction of brain images. *NeuroImage*.

[33] Yan C., Zang Y. (2010). DPARSF: a MATLAB toolbox for “pipeline” data analysis of resting-state fMRI. *Frontiers in Systems Neuroscience*.

[34] Beggs J. M., Plenz D. (2003). Neuronal avalanches in neocortical circuits. *The Journal of Neuroscience*.

[35] Clauset A., Shalizi C. R., Newman M. E. J. (2009). Power-law distributions in empirical data. *SIAM Review*.

[36] Yang H., Shew W. L., Roy R., Plenz D. (2012). Maximal variability of phase synchrony in cortical networks with neuronal avalanches. *The Journal of Neuroscience*.

[37] Peng C. K., Buldyrev S. V., Havlin S., Simons M., Stanley H. E., Goldberger A. L. (1994). Mosaic organization of DNA nucleotides. *Physical Review. E, Statistical Physics, Plasmas, Fluids, and Related Interdisciplinary Topics*.

[38] Hardstone R., Poil S. S., Schiavone G. (2012). Detrended fluctuation analysis: a scale-free view on neuronal oscillations. *Frontiers in Physiology*.

[39] Mandelbrot B. B., Wallis J. R. (1969). Robustness of the rescaled range R/S in the measurement of noncyclic long run statistical dependence. *Water Resources Research*.

[40] Chen X., Lu B., Yan C. G. (2018). Reproducibility of R-fMRI metrics on the impact of different strategies for multiple comparison correction and sample sizes. *Human Brain Mapping*.

[41] Power J. D., Barnes K. A., Snyder A. Z., Schlaggar B. L., Petersen S. E. (2012). Spurious but systematic correlations in functional connectivity MRI networks arise from subject motion. *NeuroImage*.

[42] Power J. D., Barnes K. A., Snyder A. Z., Schlaggar B. L., Petersen S. E. (2013). Steps toward optimizing motion artifact removal in functional connectivity MRI; a reply to Carp. *NeuroImage*.

[43] Koessler L., Maillard L., Benhadid A. (2009). Automated cortical projection of EEG sensors: anatomical correlation via the international 10-10 system. *NeuroImage*.

[44] Kim J. T., Cho B. H., Choi K. H. (2019). Magnetic resonance imaging versus computed tomography angiography based selection for endovascular therapy in patients with acute ischemic stroke. *Stroke*.

[45] Roli A., Villani M., Filisetti A., Serra R. (2018). Dynamical criticality: overview and open questions. *Journal of Systems Science and Complexity*.

[46] Eke A., Herman P., Kocsis L., Kozak L. R. (2002). Fractal characterization of complexity in temporal physiological signals. *Physiological Measurement*.

[47] Renart A., de la Rocha J., Bartho P. (2010). The asynchronous state in cortical circuits. *Science*.

[48] He B. J. (2011). Scale-free properties of the functional magnetic resonance imaging signal during rest and task. *The Journal of Neuroscience*.

[49] Palva J. M., Zhigalov A., Hirvonen J., Korhonen O., Linkenkaer-Hansen K., Palva S. (2013). Neuronal long-range temporal correlations and avalanche dynamics are correlated with behavioral scaling laws. *Proceedings of the National Academy of Sciences of the United States of America*.

[50] Krzemiński D., Kamiński M., Marchewka A., Bola M. (2017). Breakdown of long-range temporal correlations in brain oscillations during general anesthesia. *NeuroImage*.

[51] Tu S., Qiu J., Martens U., Zhang Q. (2013). Category-selective attention modulates unconscious processes in the middle occipital gyrus. *Consciousness and Cognition*.

[52] Kazumata K., Tokairin K., Ito M. (2020). Combined structural and diffusion tensor imaging detection of ischemic injury in moyamoya disease: relation to disease advancement and cerebral hypoperfusion. *Journal of Neurosurgery*.

[53] Kazumata K., Tha K. K., Uchino H., Ito M., Nakayama N., Abumiya T. (2017). Mapping altered brain connectivity and its clinical associations in adult moyamoya disease: a resting-state functional MRI study. *PLoS One*.

