# Supplementary material for: Faded Critical Dynamics in Adult Moyamoya Disease Revealed by EEG and fMRI
Source: Oxid Med Cell Longev. 2021 Apr 16;2021:6640108. doi: 10.1155/2021/6640108 (PMC8064775; doi:10.1155/2021/6640108)
Supplement: Supplementary Materials — Figure S1: the schematic instruction of the process of EEG data. EEG data was preprocessed and then transferred to mean frequency data to extract the cascade through event detection. The cascade size and duration were qualified to provide critical information used for clinical diagnosis. The artifact-free data was also bandpass filtered to measure the long-range temporal correlation altered in the patients. Figure S2: the EEG recording procedure. The EEG recording was started with a 5-minute eyes-closed (EC), a 5-minute eyes-open (EO) resting state, and then 30 trials of a delayed-response working memory (WM) task for around 20 minutes and ended with a procedure composed of a 5-minute EC and a 5-minute EO resting state to examine the consistency of the data recording quality. Figure S3: abnormal functional network connectivity mode of the three groups (alpha band). WM: working memory. Figure S4: Difference of dynamic (left) and static (right) mean frequencies with nonoverlapping 10 seconds' epoch of EEG data among the three groups of hemorrhagic MMD, ischemic MMD, and healthy control in the resting and working memory states. Figure S5: differences of head motion among the three groups. The mean framewise displacement (FD) derived from Jenkinson's relative root mean square (RMS) algorithm [32] was used to measure the extent of head motion. The IMMD groups exhibited the highest FD (Welch's ANOVA, p = 0.045): (IMMD(0.064 ± 0.035) > control(0.057 ± 0.017) > HMMD(0.0478 ± 0.014)). Figure S6: the correlation between head motion and Hurst exponent. Red lines indicate the linear fit. Figure S7: between-group difference of LRTC maps with the consideration of head motion effects. A, the z-statistical difference maps of LRTC between the healthy and hemorrhagic MMD groups; the positive value is termed as the stronger effect in controls. The statistical threshold is set as z > 3.29 for a voxel-wise p threshold of 0.001; the minimum cluster size for a voxel p threshold of 0.001 a [file 6640108.f1.docx]

***Supplementary Material***

**Faded critical dynamics in adult moyamoya disease revealed by EEG and fMRI**

Yu Lei, M.D., Ph.D., Yuzhu Li, Ph.D., Lianchun Yu, Ph.D., Longzhou Xu, Ph.D., Xin Zhang, M.D., Gaoxing Zheng, Ph.D., Liang Chen, M.D., PhD., Wei Zhang, Ph.D., Xiaoying Qi, Ph.D., Yuxiang Gu, M.D., Ph.D.^*^, Yuguo Yu, Ph.D.^*^, and Ying Mao, M.D., Ph.D.

**
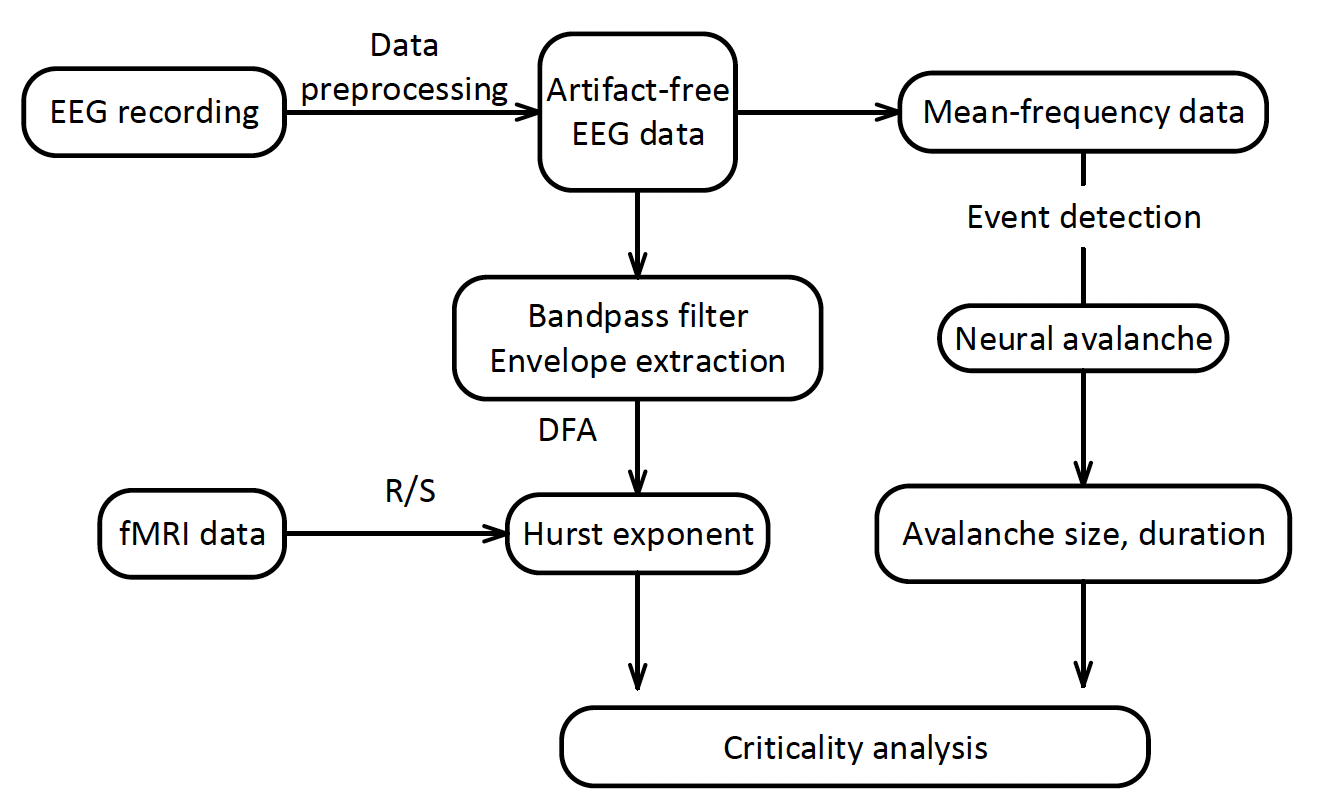
**

**Figure S1 The schematic instruction of the process of EEG data.** EEG data was preprocessed and then transferred to mean frequency data to extract the cascade through event detection. The cascade size and duration were qualified to provide critical information used for clinical diagnose. The artifact-free data was also be band pass filtered to measure the long-range temporal correlation altered in the patients.

**
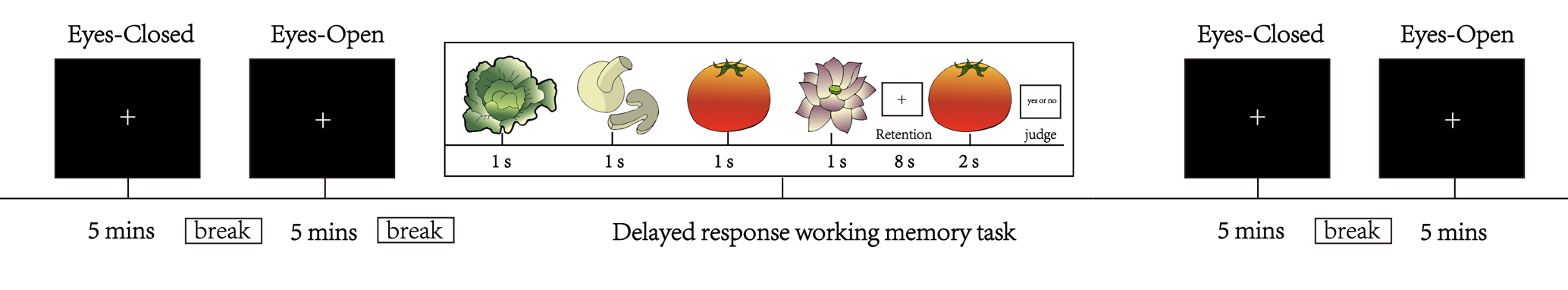
**

**Figure S2 The EEG recording procedure.** The EEG recording was started with a 5-minute eyes-closed (EC), a 5-minute eyes-open (EO) resting state, then 30 trials of a delayed-response working memory (WM) task for around 20 minutes, and ended with a procedure composed of a 5-minute EC, a 5-minute EO resting state to examine the consistence of the data recording quality.


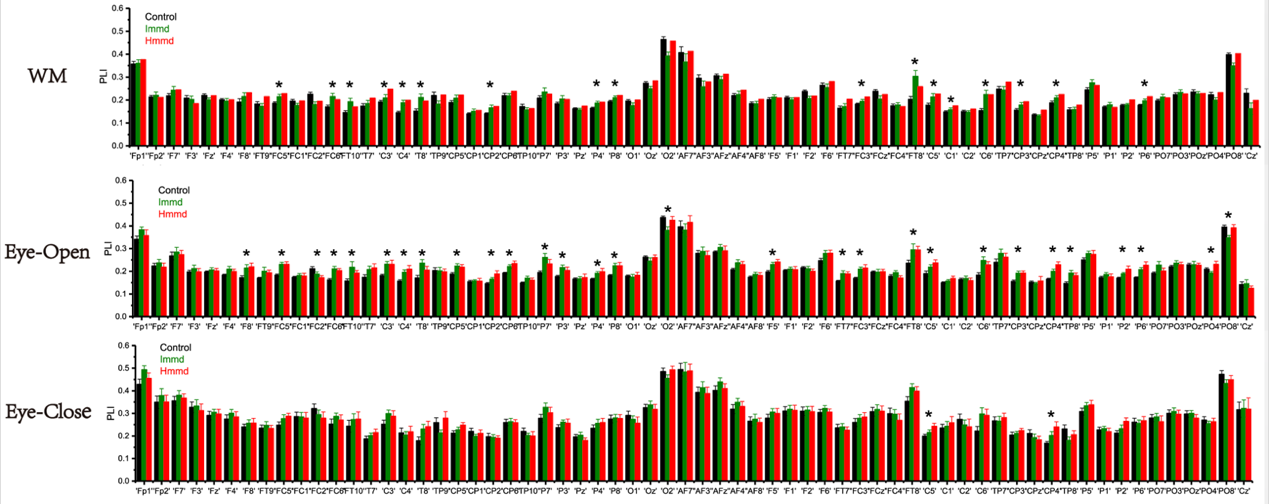


**Figure S3 Abnormal functional network connectivity mode of the three groups (alpha band).** WM, working memory.

**
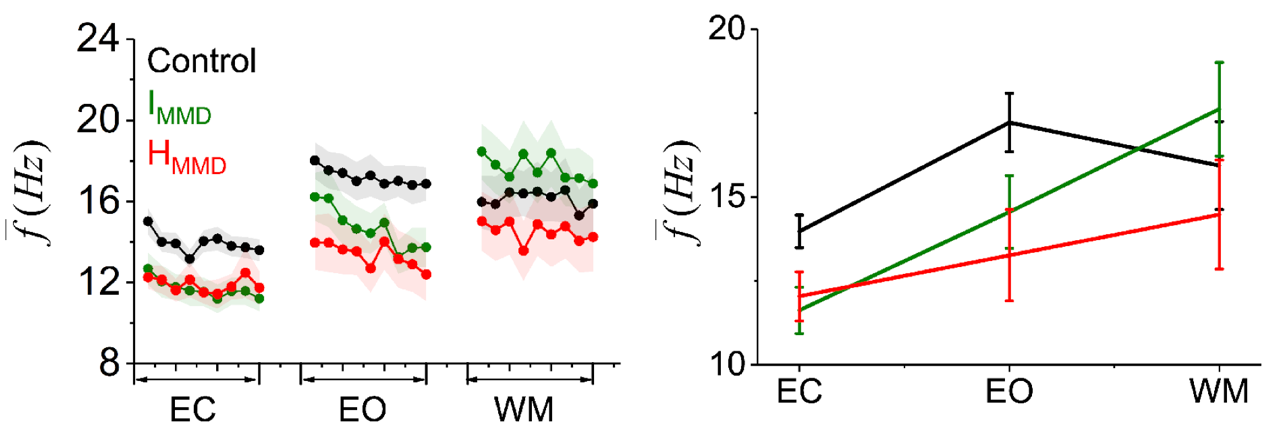
**

**Figure S4 Difference of dynamic (left) and static (right) mean frequency with non-overlap 10 seconds’ epoch of EEG data among the three groups of hemorrhagic MMD, ischemic MMD, and healthy control in the resting and working-memory states**


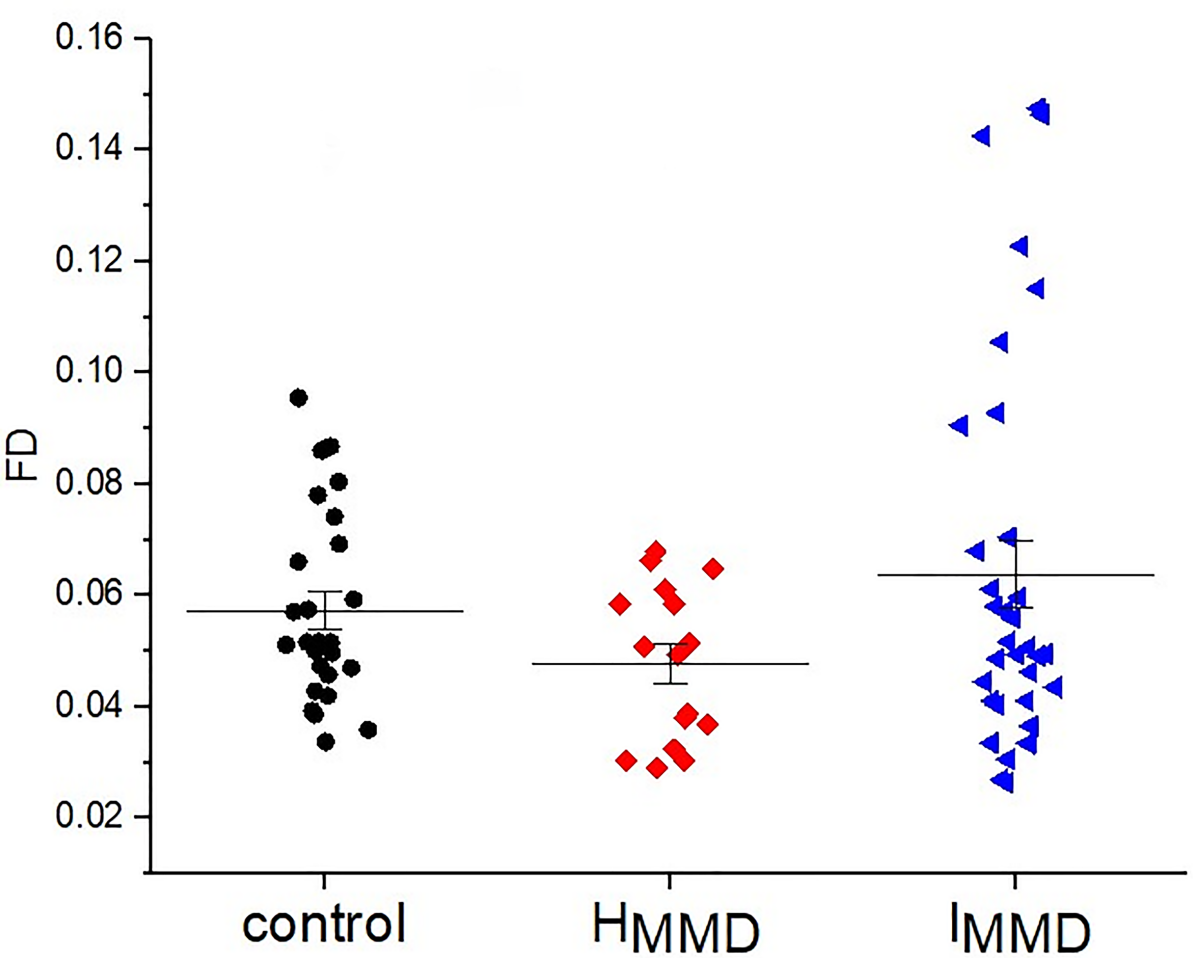


**Figure S5 Differences of head motion among the three groups.** The mean framewise displacement (FD) derived from Jenkinson's relative root mean square (RMS) algorithm (Jenkinson et al, 2002) was used to measure the extent of head motion. The I_MMD_ groups exhibited the highest FD (Welch’s ANOVA, p=0.045): (I_MMD_ (0.064 ± 0.035) > control (0.057 ± 0.017) >H_MMD_ (0.0478 ± 0.014).


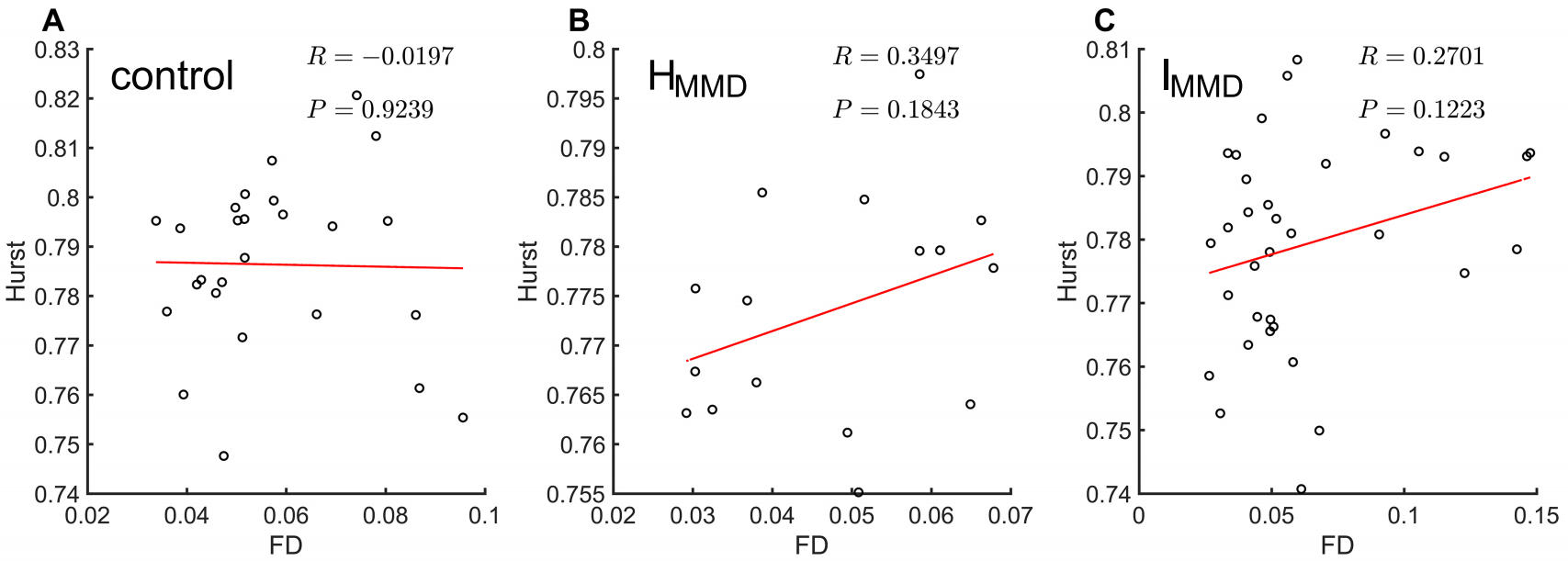


**Figure S6 The correlation between head motion and Hurst exponent.** Red lines indicate the linear fit.

**
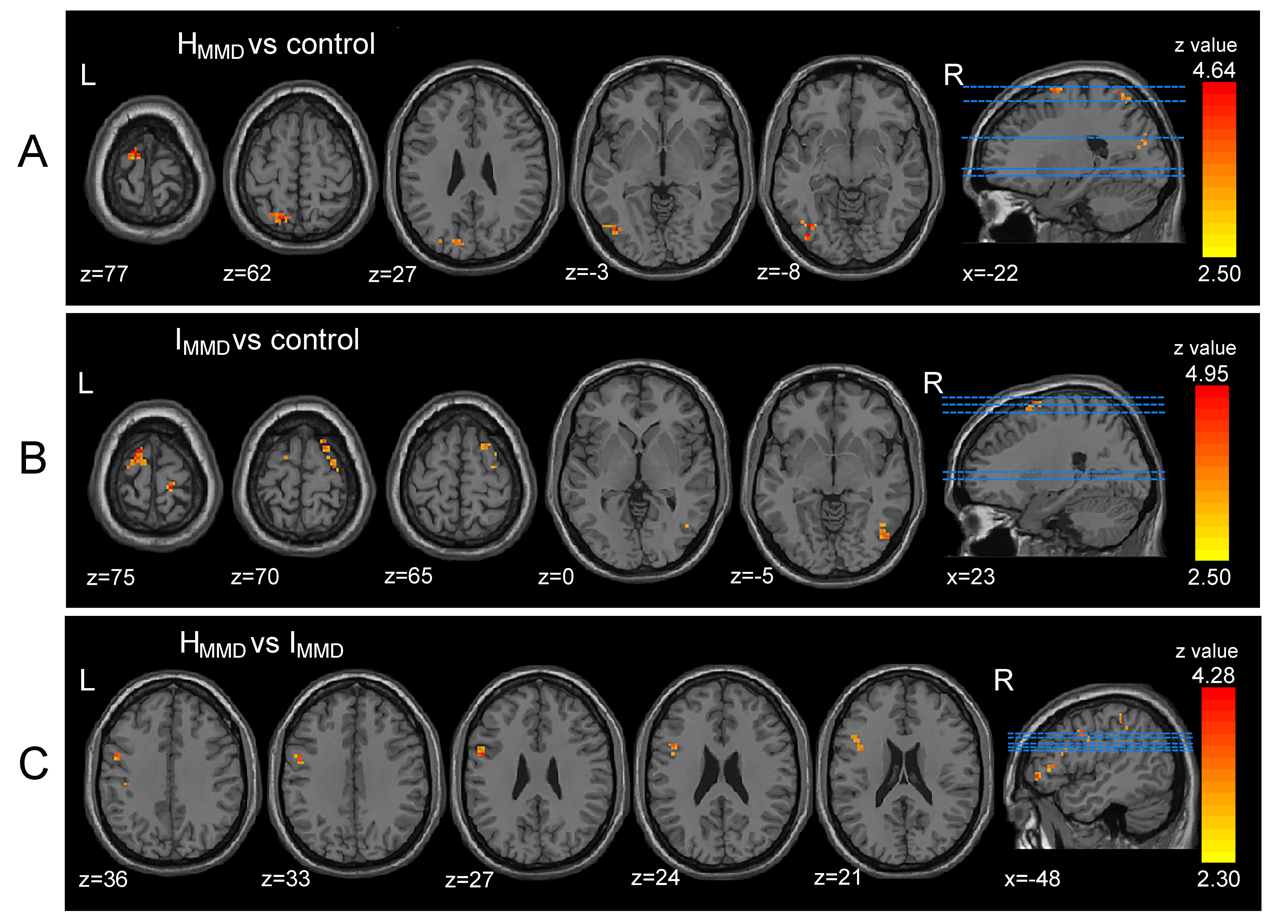
**

**Figure S7 Between-group difference of LRTC maps with the consideration of head motion effects. A**, the z-statistical difference maps of LRTC between the healthy and hemorrhagic MMD groups, positive value is termed as stronger effect in controls. The statistical threshold is set as z>3.29 for voxelwise p threshold of 0.001, the minimum cluster size for voxel p threshold 0.001 and cluster p threshold 0.05 is 24 voxels. **B**, the z-statistical difference maps of LRTC between healthy and ischemic MMD groups, positive value means stronger effect in controls. The statistical threshold is set at z>3.29 for voxelwise p threshold of 0.001, the minimum cluster size for voxel p threshold 0.001 and cluster p threshold 0.05 is 21 voxels. **C**, the z-statistical difference maps of LRTC between ischemic and hemorrhagic MMD groups, positive value indicates stronger effect in ischemic MMD. The statistical threshold is set at z>2.97 for voxelwise p threshold of 0.003, the minimum cluster size for voxel p threshold 0.001 and cluster p threshold 0.05 is 29 voxels. R, right; L, left.

**Table S1 Channels with significant difference of Hurst exponent among the three groups in all three states**

| **State** | **Channels** |
| --- | --- |
| EC | FCz, FC2, FC4, Cz, C1,O1,PO7, P7, O2, PO8, P5, P8 |
| EO | FCz, Cz, FC4, FC6, FT8, C6, T8, Pz, P2, POz, PO4, Oz, O1, O2 |
| WM | F3, FC5, FC1, FC6, C4, T8, CP2, CP6, P7, P3, P4, P8, AF7, AF3, F1, FC3, FCz, FC4, FT8, C5, C1, C2, C6, TP7, CP4, TP8, P5, P2, P6, PO7, PO3, POz, PO4, Cz |

EC, eyes-close; EO, eyes-open; WM, working memory.

**Table S2 Comparison of brain regions with significant difference in LRTC among groups with and without framewise displacement covariate**

| **T test without FD covariate** | | | **T test with FD covariate** | | |
| --- | --- | --- | --- | --- | --- |
| **Brain regions** | **Vol (mm^3^)** | **Maximum Z** | **Brain regions** | **Vol (mm^3^)** | **Maximum Z** |
| H_MMD_ vs Controls | | | | | |
| Left MOG | 783 | 4.627 | Left MOG | 567 | 4.360 |
| Left SMA | 567 | 4.533 | Left SMA | 324 | 4.640 |
| Left PCu | 324 | 4.389 | Left PCu | 216 | 4.076 |
| Left SPG | 405 | 4.402 | Left SPG | 459 | 4.237 |
| Left DLPFC | 918 | 4.311 | Left DLPFC | 648 | 4.366 |
| I_MMD_ vs Controls | | | | | |
| Left DLPFC | 675 | 5.058 | Left DLPFC | 567 | 4.950 |
| Left SMA | 459 | 4.808 | Left SMA | 432 | 4.701 |
| Right PoCG | 540 | 4.606 | Right PoCG | 243 | 4.498 |
| Right DLPFC | 972 | 4.458 | Right DLPFC | 918 | 4.408 |
| Right ITG | 702 | 4.232 | Right ITG | 675 | 4.129 |
| Right PreCG | 405 | 4.137 | Right PreCG | 351 | 4.009 |
| H_MMD_ vs I_MMD_ | | | | | |
| Left PreCG | 540 | 4.156 | Left PreCG | 621 | 3.903 |

Abbreviation: FD, framewise displacement; MOG, middle occipital gyrus; SMA, supplementary motor area; PCu, precuneus; SPG, superior parietal gyrus; DLPFC, dorsolateral prefrontal gyrus; PoCG, postcentral gyrus; ITG, inferior temporal gyrus; PreCG, precentral gyrus; IPSMAG, inferior parietal supramarginal angular gyrus.
